# Supplementary material for: FloraSyntropy-net: scalable deep learning with novel FloraSyntropy archive for large-scale plant disease diagnosis
Source: Plant Methods. 2026 Mar 17;22:35. doi: 10.1186/s13007-026-01519-4 (PMC13064331; doi:10.1186/s13007-026-01519-4)
Supplement: Supplementary file 1 — Supplementary Material 1. [file 13007_2026_1519_MOESM1_ESM.pdf]

Supplementary material

Table 14 Plant Disease Classification Public Datasets (2018-2025)

Dataset	Year	Reference)
**PlantVillage**	2018	[20]
**Cassava**	2021	[21]
**Plant Village V2**	2023	[22]
**BananaLSD**	2023	[23]
**Coffee**	2019	[24]
**Soybean**	2021	[25]
**Tea**	2022	[26]
**Sugarcane**	2025	[23]
